# Coping With Metal Toxicity – Cues From Halophytes

**DOI:** 10.3389/fpls.2018.00777

**Published:** 2018-06-19

**Authors:** Ganesh C. Nikalje, Penna Suprasanna

**Affiliations:** ^1^Department of Botany, R. K. Talreja College of Arts, Science and Commerce, Ulhasnagar, India; ^2^Nuclear Agriculture and Biotechnology Division, Bhabha Atomic Research Centre, Mumbai, India

**Keywords:** halophytes, metal toxicity, detoxification, metal transport, cross-tolerance

## Abstract

Being the native flora of saline soil, halophytes are well studied for their salt tolerance and adaptation mechanism at the physiological, biochemical, molecular and metabolomic levels. However, these saline habitats are getting contaminated due to various anthropogenic activities like urban waste, agricultural runoff, mining, industrial waste that are rich in toxic metals and metalloids. These toxic metals impose detrimental effects on growth and development of most plant species. Halophytes by virtue of their tolerance to salinity also show high tolerance to heavy metals which is attributed to the enhanced root to shoot metal translocation and bioavailability. Halophytes rapidly uptake toxic ions from the root and transport them toward aerial parts by using different transporters which are involved in metal tolerance and homeostasis. A number of defense related physiological and biochemical strategies are known to be crucial for metal detoxification in halophytes however; there is paucity of information on the molecular regulators. Understanding of the phenomenon of cross-tolerance of salinity with other abiotic stresses in halophytes could very well boost their potential use in phytoremediation. In this article, we present an overview of heavy metal tolerance in case of halophytes, associated mechanisms and cross-tolerance of salinity with other abiotic stresses.

## Introduction

Among the abiotic stresses, toxic metal stress is one of the major threats to plant growth and development. Increased industrialization has exacerbated the contamination of the soil ecosystem through increased heavy metal concentrations that are toxic and hazardous to the living organisms (Mishra et al., 2017). While plants require some heavy metals like Cobalt (Co), Copper (Cu), Iron (Fe), Manganese (Mn), Molybdenum (Mo), Nickel (Ni), Vanadium (V), and Zinc (Zn) in very minute concentrations, higher levels can be very toxic to plant growth and development. Certain heavy metals like Lead (Pb), Cadmium (Cd), Mercury (Hg), and Arsenic (As) are considered highly toxic and pose a threat to environment (Chibuike and Obiora, 2014). Increased metal accumulation in the soil leads to competition between toxic metals and essential nutrients for absorption by plants and causes excess accumulation in plants (Zhuang et al., 2014). Metal stress affects plant growth either directly or indirectly (Patra et al., 2004). Direct effects include, inhibition of cytoplasmic enzymes and causes oxidative stress and indirect effects lead to oxidative stress, generation of excess reactive oxygen species that oxidizes biomolecules and disturb ion homeostasis in the plant (Hossain et al., 2012); deplete glutathione; bind to sulfhydryl groups of proteins, and inhibit activity of antioxidant enzymes (Bielen et al., 2013). Many crop plants are sensitive to metal stress and hence are referred to as non-accumulator plants, while some plants are hyper-accumulators and can tolerate heavy metals at higher levels (Rascio and Navari-Izzo, 2011). Avoidance of metal uptake from the metalliferous soil and/or exclusion from roots itself are the strategies adopted by plants to prevent metal uptake and its movement into shoots (Viehweger, 2014).

Halophytes are the natives of saline soils rich in Na^+^, Cl^-^ and other toxic metal ions. These plant species have the ability to combat several abiotic stress constraints exerted by their natural habitat (Lutts et al., 2004). Besides salinity, halophytes exhibit high tolerance to toxic metals and can survive under high concentration of toxic metals ions where most plants do not survive (Wang et al., 2013). It has been suggested that such cross tolerance can be attributed to some kind of cross talk mechanism between salinity and metal stress (Ben et al., 2013). It is thus important to understand the biological mechanisms that enable these plants to thrive under toxic levels of salinity and heavy metals. In addition, the microbiota associated with halophyte roots, play a major role in detoxification of toxic metal ions (Vacheron et al., 2013). They affect the bioavailability of toxic ions by sequestration, precipitation and changing oxidation state of heavy metals (Kang et al., 2016). In the present article, we briefly outline the features of halophytes for phytoremediation of heavy metals in view of their metal induced responses, detoxification mechanism and the intervention of halotolerant microbiota.

## Plant Responses to Metal Stress

Increasing concentration of toxic metals in the rhizosphere alters normal physiological and metabolic processes in plants. They compete with essential nutrients for absorption from roots for example Cs, As, Cd compete with K, P and Zn respectively and cause nutrient deficiency (DalCorso et al., 2013). To counter such situation, plants have developed number of strategies to survive including sensing the increased concentration of metals, transduction and transmission of signal and triggering stress responsive elements (Sruthi et al., 2016). The plant photosynthetic machinery is highly vulnerable to metal toxicity. Cadmium severely affects chlorophyll content, photosynthetic rate and intracellular CO_2_ concentration (Dong et al., 2005). Other metals copper (Cu), manganese (Mn), nickel (Ni), and zinc (Zn) also reduce chlorophyll content as a result of decreased photosynthetic efficiency of PS II in *Elodea densa* (Maleva et al., 2012) and *Thalassia hemprichii* (Li et al., 2012). Similarly, Cd hinders RUBISCO activity by forming mercaptide with thiol group of RUBISCO in *Erythrina variegata* (Siborova, 1988; Muthuchelian et al., 2001). Copper (Cu) is inhibitor of carboxylase and oxygenase activities of RUBISCO enzyme (Lidon and Henriques, 1991). It interacts with cysteine residue of RUBISCO and decreases its activity in *Chenopodium rubrum* (Schafer et al., 1992). Over all metal toxicity leads to reduction in chlorophyll pigments, rate of photosynthesis, PS II quantum yield, stomatal conductance and assimilation of CO_2_ and causes changes at cellular and tissue levels (Singh et al., 2016).

At cellular level, chromium disturbs cell cycle, inhibits cell division, and thereby reduces root growth (Sundaramoorthy et al., 2010). Cd decreases expression of a cyclin dependant kinase (CDK) which results in to altered transition of G1 to S phase and progression of cell cycle (Pena et al., 2012). Copper (Cu) alters auxin distribution by modulating PIN1 proteins and causes inhibition of primary root elongation (Peto et al., 2011; Yuan et al., 2013). Two Zn^2+^ tolerant species of Arabidopsis, *A. halleri*, and *A. arenosa* under metals stress show low plasma membrane depolarization than metal sensitive *A. thaliana* which resulted in rapid membrane voltage changes and more metal toxicity in the sensitive species (Kenderesova et al., 2012).

Metal toxicity also hampers nitrogen metabolism, which is the vital physiological processes in growth and development of a plant. The heavy metals induce protease activity and thereby reduce activity of nitrate metabolizing enzymes such as nitrate reductase, nitrite reductase and ammonia assimilation enzymes such as glutamine synthetase, glutamine oxoglutarate aminotransferase, and glutamate dehydrogenase (Chaffei et al., 2003). Cadmium severely affects nitrate metabolism by inhibition of nitrate uptake and transportation (Lea and Miflin, 2004) ultimately leading to altered primary nitrogen assimilation process.

Plant hormones play an essential role in the control of plant growth, development and tolerance against abiotic stresses (Singh et al., 2017). They coordinate signaling mechanisms under stressful conditions and stimulate adaptive responses in halophytes (Bücker-Neto et al., 2017). Majorly ABA, Salicylic acid, Ethylene and cytokinins are linked with stress tolerance of plants (Singh et al., 2017). In a halophyte, *Kosteletzkya virginica*, under cadmium stress hormones such as ABA, 1- aminocyclopropane-1-carboxylic acid, zeatin riboside and zeatin level was increased (Han et al., 2012). The zeatin and zeatin riboside are known anti-senescing agents that functions in delay of breakdown of chlorophyll pigments and degradation of cell membrane and proteins (Sýkorová et al., 2008). In a comparative study between a halophyte, *S. chilense* and a glycophyte *S. lycopersicum*, hormonal profiling revealed that the hormones (cytokinins, ethylene and salicylic acid) showed positive co-relation with osmotic potential in former while negative correlation with osmotic adjustment in later case under salt stress (Gharbi et al., 2017). In *Cakile maritima*, methyl jasmonate and salicylic acid are shown to be involved in the amelioration of Cd induced toxicity along with osmolytes such as proline and betaine (Taamalli et al., 2015). In a metallophyte *Brassica juncea*, Srivastava et al. (2015) have shown that ‘Arsenic’ (As) stress imposes toxicity by altering levels of auxins and expression of different microRNAs. In addition, exogenous supplementation of IAA improved growth of hyperaccumulator plant, *Brassica* under ‘As’ stress which confirms role of hormones in managing ‘As’ induced alterations in plant cell (Srivastava et al., 2015). The crucial role of hormonal modulations during stress adaptation in halophytes need to be studied to understand their interactions with different pathways of signaling, defense and cross talk.

## Mechanisms of Toxic Metal Detoxification

**FIGURE 1:**
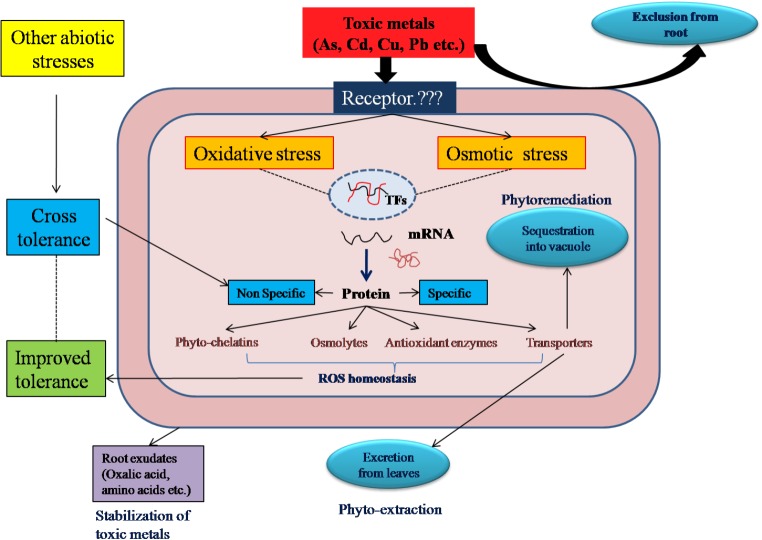
Integrative view of mechanism of toxic metal tolerance, cross-tolerance and application in phytoremediation of halophytes: To avoid over accumulation of toxic metals, halophytes employ different strategies like exclusion, excretion and accumulation to get rid of toxic metal stress. However, under high metal stress, metal ions impose both oxidative and osmotic stresses. In response to these stresses, halophytes activate cascade of molecular regulators and synthesize either non-specific or specific proteins (specific to particular metal). The non-specific proteins are involved in cross-tolerance with all other abiotic stresses. These proteins carryout ROS homeostasis and achieves metal tolerance in halophytes.

Halophytes show three biological detoxification mechanisms to combat the metal toxicity namely, metal ion exclusion, excretion and accumulation (**Figure 1**). Exclusion is the process where metal ions are selectively excluded from roots and their entry in xylem stream is restricted. *Avicennia marina*, a highly metal tolerant halophyte, shows exclusion and/or avoidance mechanism (Burchett et al., 2003). The plant selectively excludes lead (Pb) ions from roots (MacFarlane and Burchett, 2002). *Bruguiera gymnorhiza* efficiently excludes Cu and Cd ions from roots and shows high tolerance to these toxic ions (Wang et al., 2013). *Atriplex* also employs the ion exclusion mechanism for metal tolerance (Kachout et al., 2012).

In the excretion type of mechanism, plants possess special morphological features like glands, hairs, trichomes or bladders on their leaf or stem. In a halophyte, *Tamarix smyrnensis* Cd and Pb, metal tolerance is achieved by accumulating excess metal ions in salt glands and excreting them on leaf surface (Lefevre et al., 2009). *Limoniastrum monopetalum* was assessed for phytoremediation of Cd and Pb from contaminated soil. The presence of excreted crystals of Cd and Pb on leaf surface confirmed the possible mechanism of metal excretion in this halophyte (Manousaki et al., 2014). Metal excretion as the prime mechanism has also been noted in halophytes such as *Atriplex halimus, Atriplex marina, Armeria maritima*, and *Tamarix aphylla*. The salt glands of these halophytes are not specific to salt ions alone, but can secrete other toxic metal ions (Lokhande and Suprasanna, 2012) (**Figure 1**).

In the accumulation mechanism, some halophytes do not possess special morphological features and/or unable to exclude from root. They absorb toxic salt ions and rapidly translocate towards aerial parts like leaves. These ions are sequestered in to vacuoles to avoid accumulation in the cytoplasm (Nikalje et al., 2017a). Similar to salt ions, *Juncus acutus, Mesembryanthemum crystallinum, Salicornia maritime, Spartina alterniflora, Sesuvium portulacastrum* accumulate toxic metal ions in the aerial parts (Christofilopoulos et al., 2016; Pan et al., 2016) (**Figure 1**).

## Reactive Oxygen Species (ROS)

Generation of ROS is an indispensible consequence of any type of stress. The major sites of ROS production are the chloroplast, mitochondria and peroxisomes. Accumulation of heavy metals results in the disturbance of CO_2_ in chloroplast and it reduces photosynthetic electron transport chain and generation of ROS (Mittler et al., 2004). In mitochondria also, over reduction of electron transport chain leads to generation of ROS (Keunen et al., 2011). Mitochondria convert 1–5% of total O_2_ consumed in to ROS (Moller et al., 2007). In peroxisomes, during photorespiration, hydrogen peroxide is produced by oxidation of glycolate in to glyoxylic acid (Mittler et al., 2004). Thus, different ROS molecules such as singlet oxygen, superoxide anion, hydrogen peroxide and hydroxyl ions are formed during functioning of ETS (Sharma and Dietz, 2009). The metals like, Cu, Cd, Fe, Zn induce ROS production through Haber-Weiss and Fenton reactions by hampering the enzyme activities involved in antioxidant defence (Keunen et al., 2011). ROS plays a dual role in plant metabolism. Under optimum concentration they act as stress sensor and are involved in many physiological processes like cell growth, cell differentiations, root hair growth, stomatal growth and as stress sensor (Tsukagoshi et al., 2010). The fate of ROS is dependent on scavenging system. If the scavenging system efficiently scavenges ROS then it acts as signaling molecule. However, if the ROS generation is high and scavenging system fails to regulate it, then it becomes toxic (Miller et al., 2010).

## Antioxidant Defense

Antioxidant system constitutes the enzymatic or non-enzymatic antioxidant components which protects cells from damaging effects of ROS. The enzymatic antioxidants consists of superoxide dismutase (SOD), catalase (CAT), ascorbate peroxidise (APX), glutathione reductase (GR) etc. These antioxidant enzymes are involved in the mitigation of metal induced damages. It is well established that metal enhances ROS generation and significant increase in antioxidant enzymes (Bashri and Prasad, 2015). In a metallophyte, *Brassica juncea* under copper treatment POD, APX, and SOD activity was increased (Wang et al., 2004). Under Pb treatment ascorbate peroxidise, catalase, guaiacol peroxidise, superoxide dismutase enzyme activities were significantly increases and with increase in Pb concentration from 50 to 100 μM the activity was increased (Bharwana et al., 2013). In addition, *Cakile maritima*, in responce to Cd stress, plant has promoted activity of antioxidant system by high up regulation of *SOD1.* This gene plays a vital role in detoxification of metal induced toxicity as it modulated amount of reactive oxygen species such (H_2_O_2_ and O_2_) (Taamalli et al., 2015).

## Phytochelatins

Phytochelatins (PCs) are the low molecular weight polypeptides synthesized by phytochelatin synthase (PCS) enzyme from glutathione. PCs contribute to the transport of metals to vacuoles and thus contribute to detoxification. The synthesis of PCs is high energy consuming, therefore metallophyte rarely use this strategy. However, in non-metallophytes, the presence of constitutive PCs may help to reduce damage under high metal load (Tennstedt et al., 2009). In a halophyte, *Avicennia germinans*, the treatment of Cd^2+^ and Cu^2+^ highly up regulated *AvPCS* gene within hours of treatment (Gonzalez-Mendoza et al., 2007). Although this up regulation was transient, this was sufficient to activate long-term protective mechanism for detoxification of these heavy metals. It is reported that in halophytes, *Atriplex halimus* and *Suaeda fruticosa*, overproduction of phytochelatins was shown to be involved in tolerance against Cd and Zn stress (Lutts et al., 2004; Bankaji et al., 2015).

## Metallothioneins

Metallothioneins (MTs) are ubiquitous metal binding proteins, rich in cystein amino acid (Cobbett and Goldsbrough, 2002). The metal binding property of MTs is known to be crucial in metal homeostasis. There are two isoforms of metallothionein *MT1* and *MT2.* It is observed that *MT1* mainly expressed in roots and *MT2* is localized in leaves. Under Cu, Pb and Zn metal stress *Bruguiera gymnorhiza* type *MT2* gene was highly up regulated (Huang and Wang, 2009). In addition, in *Avicennia marina* over expression of *AmMT2* showed enhanced tolerance to Cd, Cu, Pb and Zn treatment (Huang and Wang, 2010). Halophytes show differential responses to different metals. In *Prosopis juliflora*, the *PjMT*-2 was up regulated by several folds under Zn treatment but its expression remain unchanged under Cd and Cu treatments (Usha et al., 2009). Under the same stress treatment, in *Salicornia brachiata* the expression of *SbMT2* was altered (up-regulated) by Zn and Cu while remained unaffected under Cd treatment (Chaturvedi et al., 2012). This indicates that halophytes are specific to certain toxic metal ions and this specificity can be utilized during selection of a halophytic species depending on the composition of toxic metal in the contaminated soil for phytoremediation.

## Metal Transporters

Halophytes rapidly uptake toxic ions from the root and transport them toward aerial parts. The root zone (rhizosphere) thus could become free and roots carry out their normal metabolism. In aerial parts, these toxic ions are transported from cytoplasm into vacuole through the action of different transporter proteins. These include, ATPases, Cation diffusion facilitator (CDF), Multidrug And Toxin Efflux (MATE), Natural Resistance-Associated Macrophage proteins (NRAMP) and Zinc-Iron Permease (ZIP) family proteins. The ATPases are further divided in to CPz- type and P1B type. The former ATPases are involved in transport of Cd, Cu, Pb, and Zn with the help of ATPases across the cell (Williams et al., 2000) whereas the later are also involved in influx and vacuolar storage. In addition, these ATPases regulate metal tolerance and homeostasis (Axelsen and Palmgren, 1998). These ATPases are highly up regulated in roots and shoots of metallophytes as compared to non- metallophytes (Papoyan and Kochian, 2004).

Heavy metal associated (HMA) protein contains two heavy metal binding domains with characteristics of heavy metal transporter proteins. The expression of ACHMA1 protein from *Atriplex canescens* in yeast resulted in improved tolerance of yeast to iron and other abiotic stresses (Sun et al., 2014). The cation transporter McHKT1 isolated from *Mesembryanthemum crystallinum* showed high similarity with HKT1 (known as sodium/potassium transporter). In *Xenopus* oocytes this protein showed high specificity with toxic metals Rubidium (Rb) than Caesium (Cs) as compared to potassium, sodium and lithium. This is a plasma membrane localized protein with possible function in ion homeostasis (Su et al., 2003). The NRAMP is a class of protein family, involved in the transport of heavy metal ions. In rice, there are three isoforms of this protein namely OsNRAMP1, OsNRAMP2 and OsNRAMP3 and in *Brassica* five isoforms BjNRAMP1, BjNRAMP2, BjNRAMP3, BjNRAMP4, BjNRAMP5 are known to be highly expressed in roots and shoot to facilitate metal transport (Belouchi et al., 1997).

The CDF proteins, also known as ‘cation efflux transports’, are involved in the transport of Cd, Co and Zn cations and efflux them out of cytoplasmic compartment (Maser et al., 2001). A member of ZIP protein family, ZNT1 is highly expressed in root, shoots of *T. caerulescens*, and facilitates transport of Zn ions (van der Zaal et al., 1999). The FDR3 protein is a member of MATE protein, which regulates metal translocation. In *Arabidopsis halleri* and *T. caerulescens* roots, this gene is highly expressed (Kramer et al., 2007). This reveals that both plants rapidly translocate metal ions from roots to shoot and keeps root zone free for normal functioning of roots.

## Cross-Tolerance and Stress Memory

Halophytes undergo various adaptations due to changing stressful environment by evolving cross-tolerance and developing stress anticipator (Dhar et al., 2013). Exposure of a plant to single stress activates the plant response, which facilitates tolerance to different types of stresses. This phenomenon is known as cross- tolerance (Foyer et al., 2016) (**Figure 1**). During cross-tolerance, two different types of pathways may activate a signaling cascade. These two different signaling pathways may operated independently result in same kind of response at the end or interact with each other to give final response (Knight and Knight, 2001). They could be additive regulatory pathway, negative or competitive pathway (Knight and Knight, 2001; Capiati et al., 2006). A halophyte, *Thellungiella salsuginea* tolerates both high salinity and oxidative stress, which illustrate about the action of cross talk between combined stresses (Taji et al., 2004). Cross talk includes elements under stress which lead to cross tolerance are stress sensors, calcium, CDPK’s, MAPKs cascade and transcription factors (Chinnusamy et al., 2004). The evolution of signaling system also involves hormone, oxidants and antioxidants that result in optimization of tolerance response to many constraints (Munne-Bosch et al., 2013). In a model halophyte, *Thellungiella salsuginea*, the genes *ThCBL* encode calcineurin B-like protein, *ThC4PT1* encode cyclophilin, *ThZF1* encodes Cys-2/His-2 transcription factor, involved in cross talk are studied for their functional validation in plants (Amtmann, 2009).

There is a difference between the kind of stress responses given by plants such as negative response, which result in stress damage, and improved response exhibiting additional sensory memory (Walter et al., 2013). After exposure to stress, plants keep a stress memory ‘imprint’ that can improve plant’s response (Bruce et al., 2007). The accumulation of transcription factor or signaling protein; epigenetic change involving chemical change at DNA (DNA methylation and acetylation), histone modification or accumulation of small RNA are considered as possible mechanisms of stress imprint (Ben et al., 2013).

Akin to animals, pre exposure to a stress condition causes enhancement of tolerance to subsequent stress imposition in plants. In *Cakile maritima*, pre-treatment with salinity has resulted in to improvement in tolerance to oxidative stress (Ben et al., 2013). The stress memory remains after several weeks or more and helps the plant to protect them against reactive oxygen species more efficiently than non-pre-exposed plants (Ellouzi et al., 2011). In stress memory, hormones like salicylic acid, abscisic acid and jasmonic acid also play important role. In *Cakile maritima* on exposure to high salt concentration, increased level of jasmoic acid resulted in strong response in correlation with low H_2_O_2_ and MDA level (Jaskiewicz et al., 2011). Stress pre-treatment alleviated salt induced oxidative stress and reduced jasmonic acid level in leaves of *Cakile* (Ryals et al., 1996). The priming effect of drought and cadmium on cellular metabolism of plants is similar to salt stress pre-exposure signifying the effective cross-tolerance response in above species (Ryals et al., 1996). The ability of organism to use its present environment to trigger gene expression, which leads to physiological changes in plants and subsequent adaptation to further change in environment, is referred as anticipation (Ben et al., 2013). The concept of anticipation and memory has significance in response of plants to biotic stresses (Ben et al., 2013). Some exogenous application of salicylic acid is trigger abiotic and biotic stress resistance mechanism with some salicylic acid analogs such as benzo (1, 2, 3) thiodiazole 7-carbothionic acid *S*-methyle aster (BTH) (Conrath, 2009).

## Transcriptomics in Halophytes

Transcriptomics aims at cataloging all the transcripts induced under specific physiological condition and quantification of modulated expression of each transcript (Wang et al., 2009). To unravel the salt adaptation mechanism in halophytes, transcriptomics of few halophytes has been attempted but the information about toxic metal detoxification and/or tolerance is limited to physiological, and biochemical level. A few metal responsive genes were also studied for their role in salt tolerance mechanism in halophytes (**Table 1**). These genes include Phytochelatins (*PCS*), Metallothionein (*MT*), plasma membrane ATPases (*PM H^+^ -ATPase*), pyrroline-5-carboxylate synthase (*P5CS*), Catalase (*CAT*) etc. It is now well established that halophytes show cross-tolerance mechanism among different abiotic stresses. In a halophyte, *Sesuvium portulacastrum*, the salt treatment improves cadmium tolerance (Mariem et al., 2014). Other halophytes also show tolerance to both salinity and metal stress for example *Mesembryanthemum crystallinum* tolerates copper (Thomas et al., 1998), *Atriplex halimus* tolerated lead and cadmium (Manousaki and Kalogerakis, 2009). There are commonalities between metal and salt stress, both cause production of excess reactive oxygen species (ROS) and synthesis of osmolytes and activation of antioxidant system (Sruthi et al., 2016). Although there is no report on halophyte transcriptomics under metal stress, clues about cross-tolerance related genes can be derived from transcriptomics under salt stress (**Table 2**). Mostly the ROS detoxification related genes are shown to be highly up regulated in all the studies. In *Karelinia caspica, Halogeton glomeratus, and Ipomoea imperati* ABA signaling genes were up regulated (Zhang et al., 2014; Wang et al., 2015; Luo et al., 2017). This indicated that they follow ABA dependant pathway of ion homeostasis. Other halophytes like *Porteresia coarctata*, *Caragana korshinskii, Avicennia officinalis* and *Ipomoea imperati* (Garg et al., 2013; Li et al., 2016; Krishnamurthy et al., 2017; Luo et al., 2017) showed induction of transcription factors like *MYB, AP2-EREBP, bZIP, NAC* which may interact with their downstream targets to activate the tolerance mechanism. These studies can only help to identify the cross-tolerance related gene(s) but there is need to establish the transcriptomic networks under metal stress in halophytes.

**Table 1:** Gene expression studies in halophytes under metal stress.

Halophyte	Treatment	Up regulated gene(s)	Reference
*Aeluropus littoralis*	Ag, Hg, Pb	*PMH^+^ -ATPase*	Jam et al., 2014
*Avicennia germinans*	Cd – Cu	*AvPCS*	Gonzalez-Mendoza et al., 2007
*Avicennia marina*	Zn, Cu, Pb	*AmMT2*	Huang and Wang, 2010
*Bruguiera gymnorrhiza*	Zn, Cu, Pb	*BgMT2*	Huang and Wang, 2009
*Mesembryanthemum crystallinum*	Cu	*HSP60*	Thomas et al., 1998
*Paspalum vaginatum*	Cd	*PCS1, PCS2, CYP450, HSFA4a, UGP*	Chen et al., 2016
*Salicornia brachiata*	Zn, Cu, Cd	*SbMT*-2	Chaturvedi et al., 2014
*Suaeda salsa*	Cd	Phytochelatin synthase, *CAT2*	Cong et al., 2013

**Table 2:** Transcriptomics studies in halophytes (under salt stress) reveal involvement of cross-tolerance related genes in toxic metal tolerance.

Halophyte	Platform used	Up-regulated cross tolerance related genes	Reference
*Avicennia officinalis*	Illumina HiSeq^TM^ 2000	*ERF, MYB, bZIP*, Cadmium ion responsive genes	Krishnamurthy et al., 2017
*Caragana korshinskii*	Illumina HiSeq 2000	*SOD, CAT, APX, POX, MYB, NAC, ERF*	Li et al., 2016
*Halogeton glomeratus*	Illumina HiSeq2000	*POD, GPX*, ABA responsive genes	Wang et al., 2015
*Ipomoea imperati*	Illumina Hiseq. 2500	*MYB, HD-ZIP*, ABA signaling	Luo et al., 2017
*Karelinia caspica*	Illumina HiSeq 2000	ABA responsive genes	Zhang et al., 2014
*Mesembryanthemum crystallinum*	Illumina Genome Analyzer IIx	*P5CS1*	Tsukagoshi et al., 2015
*Porteresia coarctata*	Illumina Genome Analyzer II	*MYB, AP2-EREBP, bZIP, NAC*	Garg et al., 2013
*Reaumuria trigyna*	Illumina HiSeq^TM^ 2000	*GPX*, *APX*, *PODs*, *SODs*	Dang et al., 2013
*Spartina alterniflora*	Roche’s 454 GS-FLX	*ARF, MYB, H^+^-ATPase and vacuolar H^+^-ATPase CDPK*	Bedre et al., 2016
*Sporobolus virginicus*	Illumina HiSeq 2500	*P5CS*, *ERF*, *bZIP*, *MYB*, *NAC*	Yamamoto et al., 2015
*Suaeda fruticosa*	Illumina Hiseq 2000	*APX*, cadmium resistance 2–like, Aluminum–activated malate transporter 10, Magnesium transporter *NIPA2*, Vacuolar Iron transporter family,	Diray-Arce et al., 2015
*Suaeda maritime*	IlluminaHiSeq 2000	*Cu/Zn-SOD, MDHAR, Mn-SOD, Fe-SOD*	Gharat et al., 2016
*Suaeda glauca*	Illumina HiSeq 2500	Oligopeptide transporters, *APX*, *POX*	Jin et al., 2016

## Rhizobacteria – Partners in Protection

Soil microbes possess geo-active action which helps them to detoxify toxic metals (Long et al., 2002). These microbes are mainly species of *Arthrobacter*, *Bacillus* and *Pseudomonas* (Pires et al., 2017). *Rhizobium* is a nitrogen fixing, plant growth promoting bacteria and the process of nodulation and activity of nitrogenase activity are sensitive to metal stress. However, some heavy metal tolerant strains of *Rhizobium* are also identified. The Legume and *Rhizobium* association is well known for detoxification of heavy metal induced toxicity (Checcucci et al., 2017). Fungi belong to Ascomycota and Basidiomycota are common in metal contaminated soils (Narendrula-Kotha and Nkongolo, 2017). In addition, arbuscular mycorrhizal (AM) fungi were also reported from metal contaminated and nutrient poor soils (Khan et al., 2000). These microbes interact with metals and carry out metal speciation, dissolution, toxicity, mobility and deterioration (Kong and Glick, 2017). The salt marsh halophytes provide organic substances to rhizospheric microbes and show symbiotic association. A halophyte, *Spartina maritime* is colonized by sulfate reducing bacteria and thereby develops metal tolerance (Otero and Macías, 2002a,b). The halophyte, roots influence the extracellular enzymatic activity of hydrolytic enzymes like phenol oxidase, acid phosphatase etc (Reboreda and Caçador, 2008). This extracellular enzyme activity is involved in organic matter recycling and metal speciation. The uptake of metals varies with different forms of metal. In *Spartina alterniflora* and *Spartina patens* the uptake of arsenic differs with species/form of it. In addition, it differs from distribution/ accumulation in different organs. The Inorganic form of arsenic is restricted in root while other form is rapidly translocated toward shoot (Carbonell-Barrachina et al., 1998).

## Metal Detoxification Through Rhizosperic Microbes

The Metal Tolerant Microbes (MTM) alleviate adverse effects of metal stress by modulating plant growth and enhances bioavailability of metals by altering physico-chemical properties of soil, which trigger detoxification, and removal of toxic metals from soil. The alteration in the bioavailability of metals in soil is achieved by redox reactions, acidification, precipitation and complexation (Seneviratne et al., 2017). MTM releases organic acids like acetic, gluconic, oxalic, malic that lowers the pH of soil and subsequently sequesters soluble metal ions (Turnau and Kottke, 2005). *Beauveria caledonica* colonizes with mycorrhizal fungi which secretes oxalic acid and citric acid to carry out solubilization of Cd, Cu, Pb, Zn ions (Gadd et al., 2014). Wood rotting fungi like *Formitopsis* cf. meliae and *Ganoderma* aff. *Steyaertanum* produces oxalate crystals and transforms toxic metals into less toxic forms i.e from zinc sulfate, copper sulfate, cadmium sulfate, lead nitrate into zinc oxalate dehydrate, copper oxalate hydrate, cadmium oxalate trihydrate and lead oxalate respectively.

Under metal stress, plant roots secrete certain chemicals (root exudates) which are involved in changing metal bioavailability. These exudates forms metal complex and provides nutrition to colonizing microbes. In return, these microbes support survival and growth under metal stress (Kong and Glick, 2017). Root exudates consist of different amino acids, organic acids and phytochelatins (PCs) which function as intracellular binding compounds with metals. Along with root exudates, protons (H^+^) and enzymes carry out acidification in rhizosphere enhances bioavailability of metals (Ma et al., 2016).

## Halophytes in Environmental Clean Up

Halo flora could be exploited to grow them in soils challenged with heavy metals (Nikalje et al., 2017b). Halophytes have potential to be useful as ‘green technology candidates’ in phytoremediation efforts. It is cost effective because halophytes can grow in poor quality, low fertile soil and marginal land. The halophytes with exclusion or extraction ability can be utilized in phytostabilization purpose (**Figure 1**). Such halophytes, with high biomass and rapid growth will restrict the entry of toxic ions in root, will form a vegetation cover and maintain low level of toxic metals in soil. In addition, will restrict the entry of toxic ions in ground water and minimize the water and soil erosion. Lutts et al. (2016) showed that salinity influenced biosorption ability of roots of halophyte, *Kosteletzkya pentacarpos* and could provide a valuable biological material for heavy metal retention. Halophytes like *Atriplex halimus, Atriplex nummularia, Mesembryanthemum crystallinum, Sesuvium portulacastrum, Tamarix smyrnensis, Salicornia sp.* have proved their potential in phytoremediation (Lutts and Lefevre, 2015). At the laboratory level, all of them have proved as better systems for phytoremediation and should be explored at field level. In addition, halophytes possess phytoextraction ability. As halophytes are native to saline soils and grow in saline water, cultivated halophytes can be irrigated with saline/brackish/poor quality water, which otherwise cannot be used for conventional crop irrigation (Rozema and Flowers, 2008). This property will be useful to conserve drinking water and utilized for other purposes. Salinity increases bioavailability of metals in soil and promotes translocation of metals from root to shoot (Wahla and Kirkham, 2008). The *Sesuvium portulacastrum* showed higher ability of bioaccumulation of salts and heavy metals (chromium, cadmium, copper, zinc, sodium and chloride) from tannery effluent (Ayyappan et al., 2016). Plants will accumulate toxic metals in aerial parts which can be harvested easily and the phyto-remediated soil will be devoid of or lessened with soil contaminants. Rabhi et al. (2010) had cultivated *Sesuvium portulacastrum* in artificially saline soil and after phytoremediation successfully cultivated *Hordeum vulgare.* Muchate et al. (2016), further extended these results and demonstrated phytoremediation potential of *Sesuvium* at small experimental field (ECe of saline soil was decreased from 7.1 to 4.9 ds/m). Some halophytes possess special glands on leaf surface, which excrete excessive salt ions. A halophyte *Tamarix smyrnensis* excreted both metal ions (Cd and Pb) and salt ions from salt glands. This suggests that halophytes use the same set of morphological adaptations for both salt and metal ions. The excreted metal can be collected before it re-enters in to the soil and thereby reducing the metal load (Manousaki and Kalogerakis, 2009).

## Conclusion

Being highly tolerant and native flora of saline ecosystem, halophytes are well studied for their salt adaptation mechanism. These plants not only survive under salinity but can cope up with heavy metal and other stresses. Halophytic habitats are enriched with not only excess salt ions but also other toxic metal ions. It has been proved that some halophyte species can thrive under both high salt and toxic metal conditions. The phenomenon of cross-tolerance plays a vital role for halophytes to combat with both stress conditions. However, the mechanism of metal tolerance in halophytes is still unclear. Comparative account of metallophytes and halophytes will provide valuable information about key traits involved in metal tolerance and detoxification. Recent transcriptomics studies have added more insights in to genetic regulation of high salt tolerance ability in halophytes but more studies are needed to understand the molecular regulators associated with detoxification mechanisms. In addition, identification of key metal responsive genes will help to develop metal tolerant crop varieties by using biotechnological approaches. This information gateway about metal detoxification and defense pathways will help to develop strategies in other plants, and utilize halophytes for environmental clean-up and rehabilitation of contaminated soils.

## Author Contributions

GN wrote the manuscript. PS conceived, rewritten, and finalized the manuscript for publication.

## Conflict of Interest Statement

The authors declare that the research was conducted in the absence of any commercial or financial relationships that could be construed as a potential conflict of interest.
